# Genetic Modification of Mesenchymal Stem Cell to Overexpress CXCR4 Enhances Treatment Efficacy for Brain Injury After Cardiopulmonary Resuscitation

**DOI:** 10.1111/cns.70621

**Published:** 2025-09-22

**Authors:** Yongfei Liu, Li Zhang, Jingxiang Wang, Yuan Qin, Liang Zhang, Anlin Yue, Zhongting Wang, Xiao Xiao, Shuang Wang, Lu Huang, Changjun Gao

**Affiliations:** ^1^ Department of Anesthesiology Tangdu Hospital, the Fourth Military Medical University Xi'an Shaanxi China; ^2^ Department of Anesthesiology Xi'an No. 3 Hospital, the Affiliated Hospital of Northwest University Xi'an Shaanxi China

**Keywords:** asphyxial cardiac arrest, CXCR4, exosomes, mesenchymal stem cell, pyroptosis

## Abstract

**Aim:**

To investigate whether genetically modifying human umbilical cord‐derived mesenchymal stem cells (MSC) to overexpress the CXCR4 receptor can enhance their therapeutic efficacy for treating brain injury following cardiac arrest (CA).

**Methods:**

MSC were engineered to overexpress CXCR4 (CXCR4‐MSC) via lentiviral transduction. The migration capacity of these cells was tested using in vitro chemotaxis assays. In a rat model of CA/CPR, the homing ability of CXCR4‐MSC to the brain was tracked in vivo, and their therapeutic effects on neuronal death and neurological recovery were assessed. The role of exosomes and their impact on key proteins (NLRP3, ASC, GSDMD) in the pyroptosis pathway was also investigated.

**Result:**

CXCR4 overexpression significantly enhanced the migration of MSC in vitro and their homing to injured brain tissue in vivo. Treatment with CXCR4‐MSC markedly reduced neuronal death and improved neurological recovery in resuscitated rats. This was accompanied by decreased expression of NLRP3. Furthermore, exosomes derived from CXCR4‐MSC were found to suppress pyroptosis‐related proteins (NLRP3/ASC/GSDMD) in post‐CPR neurons, an effect that was reversed upon exosome inhibition.

**Conclusion:**

Genetic modification to overexpress CXCR4 enhances the therapeutic efficacy of MSC for CA‐induced brain injury by promoting their migration to the brain via the CXCL12/CXCR4 axis. A key mechanism of this protection is exosome‐mediated inhibition of neuronal pyroptosis.

## Graphical abstract

CXCR4 Gene‐Modified Human Umbilical Cord Mesenchymal Stem Cells Enhance Brain Tissue Migration in Post‐Cardiopulmonary Resuscitation Rats and Improve Neurological Outcomes by Suppressing Neuronal Pyroptosis via Exosome Secretion.

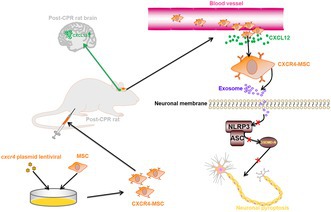

## 1 Introduction

In the perioperative period, cardiac arrest (CA) is a critical complication that results in systemic hypoperfusion, often leading to death quickly. Even with successful resuscitation, many patients still suffer from substantial neurological dysfunction. Statistics indicate that approximately two‐thirds of post‐cardiac arrest deaths result from brain injury after the restoration of voluntary circulation [1]. Furthermore, survivors may experience different levels of behavioral and cognitive dysfunction [2]. The occurrence of cerebral ischemia and hypoxia injury during CA, followed by reperfusion injury post‐resuscitation, triggers widespread neuronal damage including apoptosis, necrosis, and pyroptosis, ultimately causing various neural function deficits [3, 4]. Previous studies have demonstrated that pro‐inflammatory factors IL‐1, IL‐6, and TNF‐α are highly expressed in the brain tissue of rats after resuscitation [5]. The reduced expression of these inflammatory factors has been observed to enhance the recovery of brain function.

Mesenchymal stem cell (MSC) can decrease pro‐inflammatory cytokine release, increase anti‐inflammatory cytokine production, inhibit inflammatory cell adhesion to the endothelium, promote endothelial regeneration, and prevent cell apoptosis [6]. Due to its potent immune regulatory and anti‐inflammatory properties, the use of MSC has emerged as a promising treatment approach for organ dysfunction. However, the limited homing of intravenously injected MSC to the target tissue of inflammation significantly diminishes their therapeutic efficacy. Intravenous administration is simpler and less invasive than direct implantation. However, it results in significant MSC entrapment in the lungs and low accumulation in the ischemic brain [7]. Furthermore, while a small number of MSC may accumulate in the ischemic brain lesion after systemic infusion, they exhibit poor survival in inflammatory and hypoxic conditions. Intracerebral implantation is invasive and may cause additional damage to normal brain tissue [8].

Chemotactic factor (CF) is a low‐molecular‐weight secreted protein that mainly regulates the migration of white blood cells to injured sites [9]. C‐X‐C chemokine receptors, part of the G protein‐coupled receptor family, are commonly found in inflammatory diseases. They play a key role in controlling the migration of neutrophils and monocytes by binding with chemokines (C‐X‐C motif) ligand 12 (CXCL12), a member of the CXC family [10]. Its main receptor is CXCR4. In cases of cerebral ischemia, there is a notable increase in CXCL12 expression in and around the infarct area, leading to the recruitment of cells to the ischemic brain area through the CXCR4 pathway [11]. CXCL12/CXCR4 is important in mobilizing stem and progenitor cells from the bone marrow to the peripheral blood during ischemic myocardial injury [12]. The findings indicate that the CXCL12/CXCR4 pathway may be significant in ischemia–reperfusion injury of vital organs. Research demonstrates that genetically modified MSC can enhance their migration capability, allowing more of them to reach the intended target [13].

In our previous preliminary experiment, we observed high expression of CXCL12 in the brain tissue of rats following cardiopulmonary resuscitation (CPR). Based on this finding, we genetically modified MSC by introducing the *cxcr4* gene to promote increased expression of CXCR4. This modification was aimed at enhancing the homing ability of MSC, with the goal of improving their migration to the site of brain injury following CPR. Ultimately, the objective was to reduce neuronal injury and enhance prognosis through this targeted approach.

## 2 Result

### 2.1 The Expression of CXCL12 Increased in Brain Tissue After CPR

**FIGURE 1:**
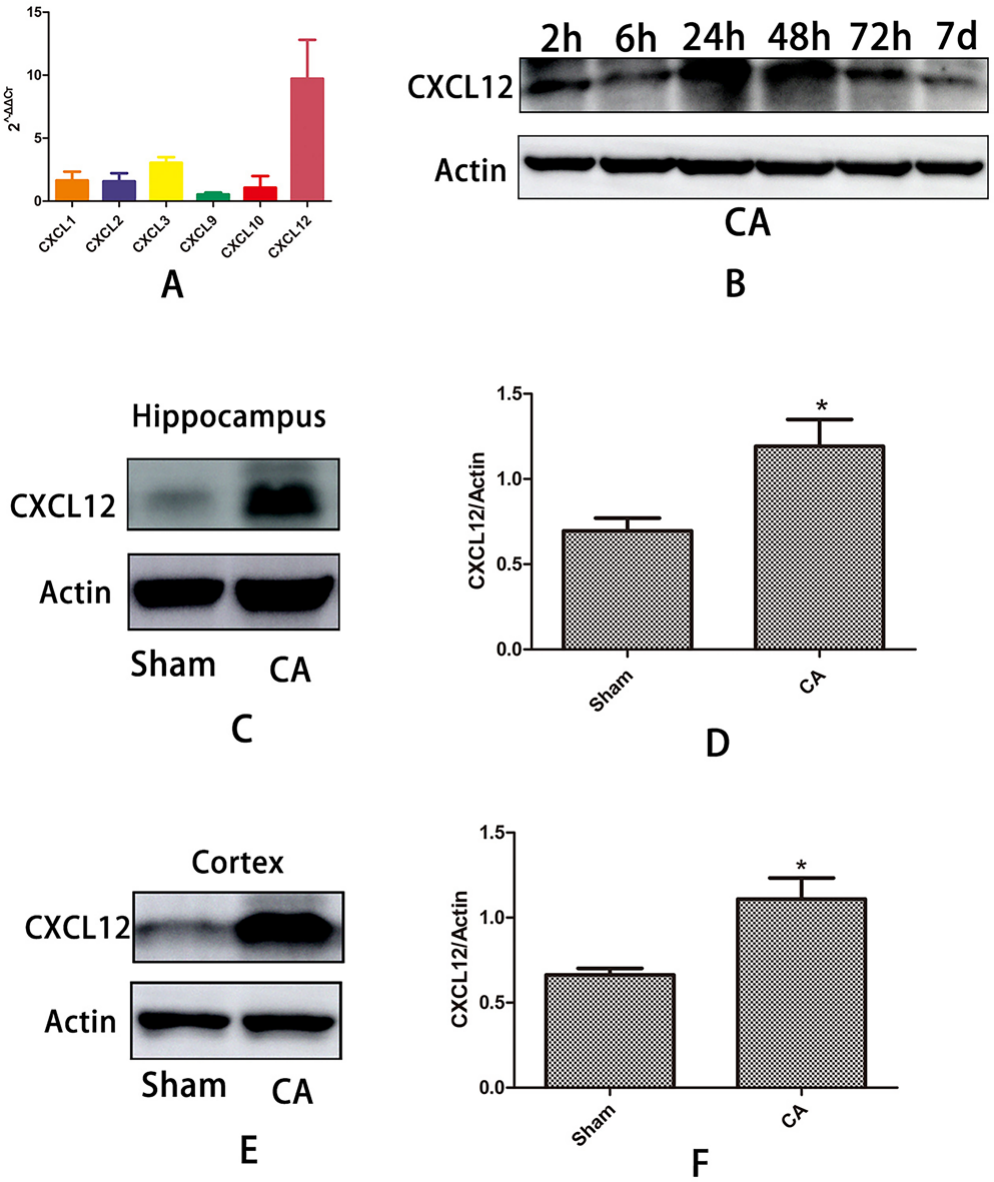
Differential expression of chemokines in the rat brain following CPR (A) The expression level of chemokine cxcl12 in brain tissue after CPR was higher than that of cxcl1, cxcl2, cxcl3, cxcl9, cxcl10, and cxcl12. (B) The expression of CXCL12 in the brain tissue of rats at 2, 12, 24, 48, 72 h, and 7 d after cardiopulmonary resuscitation. (C, D) Western blotting of CXCL12 in the hippocampus 24 h after cardiopulmonary resuscitation. (E, F) Western blotting of CXCL12 in the cortex 24 h after cardiopulmonary resuscitation.

After conducting fluorescence quantitative PCR analysis, the expression levels of chemokine RNA in rat brain tissue indicated that CXCL12 expression was the most prominent 24 h post‐CPR (Figure 1A). It was observed that CXCL12 levels in rat brain tissue began to increase as early as 2 h after cardiopulmonary resuscitation (CPR) and remained elevated at 24 h. Expression persisted until 48 , 72 h, and even at 7 days post‐CPR (Figure 1B). Subsequently, we examined CXCL12 expression in the rat hippocampus and cortex at the 24‐h time point. We observed significantly higher CXCL12 protein levels in the hippocampus compared to the Sham group (*p* = 0.017) (Figure 1C,D). A similar increase was also detected in the cortex (*p* = 0.006) (Figure 1E,F). These findings indicate that CXCL12 is the dominant chemokine expressed in brain tissue following CPR. Following culture, digestion, and passaging, human umbilical cord mesenchymal stem cells (MSC) were successfully cultured and identified through morphological assessment, surface marker analysis, and adipogenic, osteogenic, and chondrogenic differentiation potential evaluation (Figure S1).

### 2.2 Overexpression of CXCR4 in MSC Retains the Characteristic of Human MSC and Enhances Migration Potential InVitro and In Vivo

**FIGURE 2:**
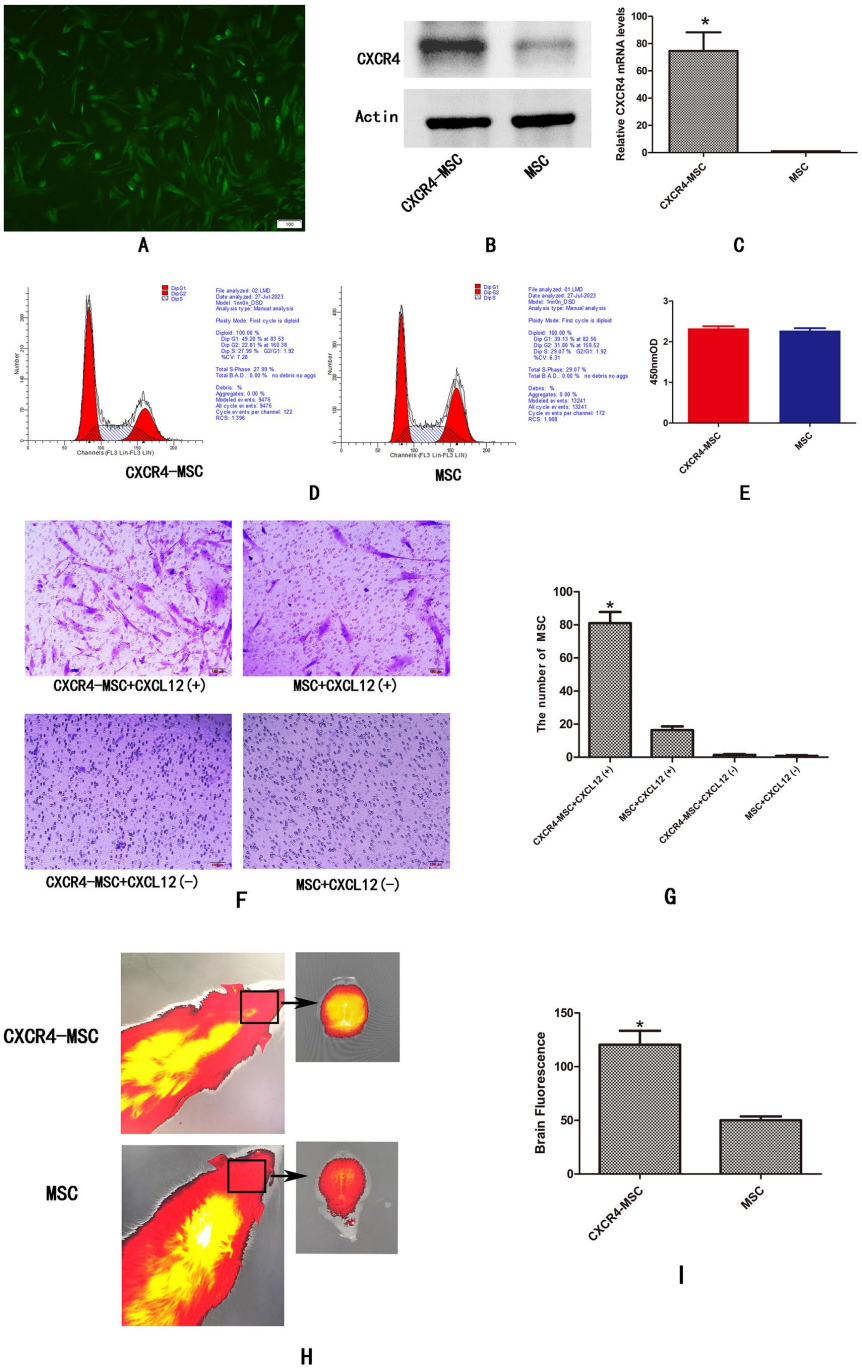
Construction of CXCR4‐MSC and its value‐added activity, cell cycle, and in vivo and in vitro migration ability. (A) Human umbilical cord MSC with fluorescent labels overexpressing CXCR4 (CXCR4‐MSC). (B) Western blotting of CXCR4 in the CXCR4‐MSC and MSC lysates. (C) The expression levels of *cxcr4* mRNA in CXCR4‐MSC and MSC after gene transduction were analyzed via RT‐qPCR. *p < 0.05. (D) The cell cycle after transfection was detected by flow cytometry. (E) Cell proliferation activity was detected by the CCK8 method. (F) Transwell assay detected the migration ability of stem cells in each group in vitro. (G) The number of migrated cells in each group. *p < 0.05 compared with the other group. (H, I) The migration ability of CXCR4‐MSC and MSC in vivo was detected in the brain of rat after CPR. *p < 0.05.

72 h after successful transfection, green fluorescence was observed in the lentivirus‐concentrated human umbilical cord MSC (Figure 2A). Real‐time fluorescent quantitative PCR and WB analysis revealed a significant increase in *cxcr4* mRNA and protein expression in human umbilical cord MSC transfected with *cxcr4* compared to normal stem cells (Figure 2B,C, *p = *0.006*). Additionally, analysis indicated that human umbilical cord MSC transfected with *cxcr4* did not exhibit any notable changes in the cell cycle compared to normal human umbilical cord MSC (Figure 2D). These observations collectively suggest that transfection with CXCR4 does not impact the growth characteristics and cell cycle of human umbilical cord MSC (Figure 2E). Subsequently, we conducted in vivo and in vitro experiments to observe the migration ability of CXCR4‐MSC. The findings from Transwell experiments demonstrated that the number of cells within the CXCR4 + MSC‐CXCL12(+) group that migrated through chemoattractant containing 100 ng/mL CXCL12 was notably higher than that observed in the MSC + CXCL12(+) group Figure 2F,G *p < 0.001. This suggests that the modification of the *cxcr4* gene in MSC can effectively enhance its chemotactic capabilities. Following this, we proceeded to transplant the *cxcr4*‐modified human umbilical cord MSC into rats post‐CPR, and utilized in vivo imaging in small animals to track their homing specifically to brain tissue. Notably, the results indicated a significant clustering of *cxcr4*‐modified stem cells within brain tissue as compared to the MSC groups (Figure 2H,I *p* = 0.004).

### 2.3 Stem Cell Modified With the *cxcr4* Gene Reduced Neurological Damage and Decreased Pyroptosis‐Related Proteins Expression

**FIGURE 3:**
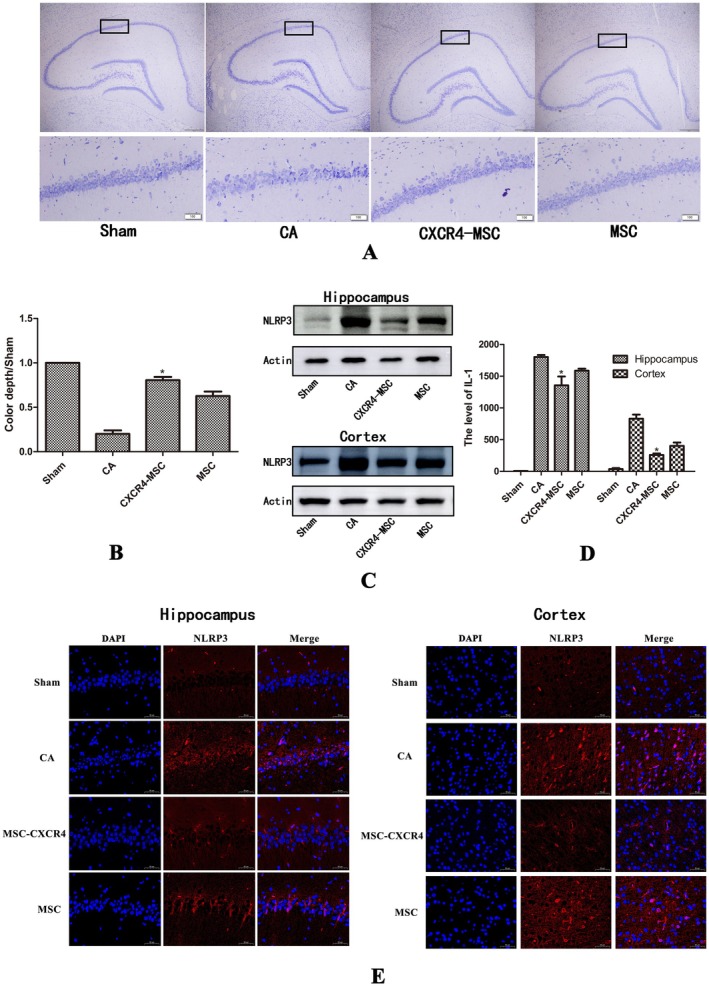
Changes of Nissl staining and pyroptosis‐related protein after resuscitation in Sham, CA, CXCR4‐MSC, and MSC treatment groups (A) Hippocampus subjected to Nissl staining. (B) Nissl corpuscle ratio in the hippocampus. *p < 0.05 compared with the CA and MSC groups. (C) Expression of pyroptotic protein NLRP3 in the hippocampus and cortex after resuscitation. (D) Expression of inflammatory factors IL‐1 in the hippocampus and cortex after resuscitation. *p < 0.05 compared with the CA and MSC treatment groups. (E) Immunofluorescence results demonstrated the expression levels of NLRP3, a pyroptosis‐related protein, in the hippocampus and cortex of brain tissues across each group of rats.

It was observed that CXCR4‐MSC treatment mitigated CA‐induced neuronal damage, as evidenced by Nissl staining indicating hippocampal cell death. Comparatively, the arrangement disorder of hippocampal cells in the CXCR4‐MSC group was significantly reduced in comparison to MSC groups, contributing to an increase in the number of surviving neurons (Figure 3A,B *p = 0.04). To verify whether the therapeutic effect was due to enhanced CXCR4‐MSC migration, we added an AMD3100 group, in which AMD3100 (a CXCR4 inhibitor) was administered intraperitoneally to the CXCR4‐MSC group. The number of stem cells migrating to the brain tissue was significantly reduced in the AMD3100 group (Figures SA and SB). Nissl staining revealed more severe neuronal damage in the AMD3100 group compared to the CXCR4 treatment group. As shown below, neuronal damage was also significantly aggravated in the treatment group after the injection of the antagonist (Figures SC and SD), indicating that the protective effects primarily stem from MSC migration. After CPR, Western blotting analysis revealed decreased expression levels related to the pyroptosis‐related protein NLRP3 in the hippocampus and cortex of rats. The expression levels in the CXCR4‐MSC group and the MSC group were found to be lower compared to those in the CA group (Figure 3C). Furthermore, it was observed in immunofluorescence that the CXCR4‐MSC group demonstrated even lower expression levels of pyroptosis‐related proteins than the MSC group (Figure 3E). ELISA showed that the expression of the pyroptosis‐related protein IL‐1 was lowest in CXCR4‐MSC compared with CA and MSC groups in the hippocampus (*p = 0.04, vs MSC) and cortex (*p = 0.036, vs MSC) (Figure 3D). These findings suggest that CXCR4‐MSC exhibit a more effective inhibition of pyroptosis of cells in brain tissue.

### 2.4 CXCR4‐MSC Inhibit Neuronal Pyroptosis in Brain Tissue After CPR by Secreting Exosomes

**FIGURE 4:**
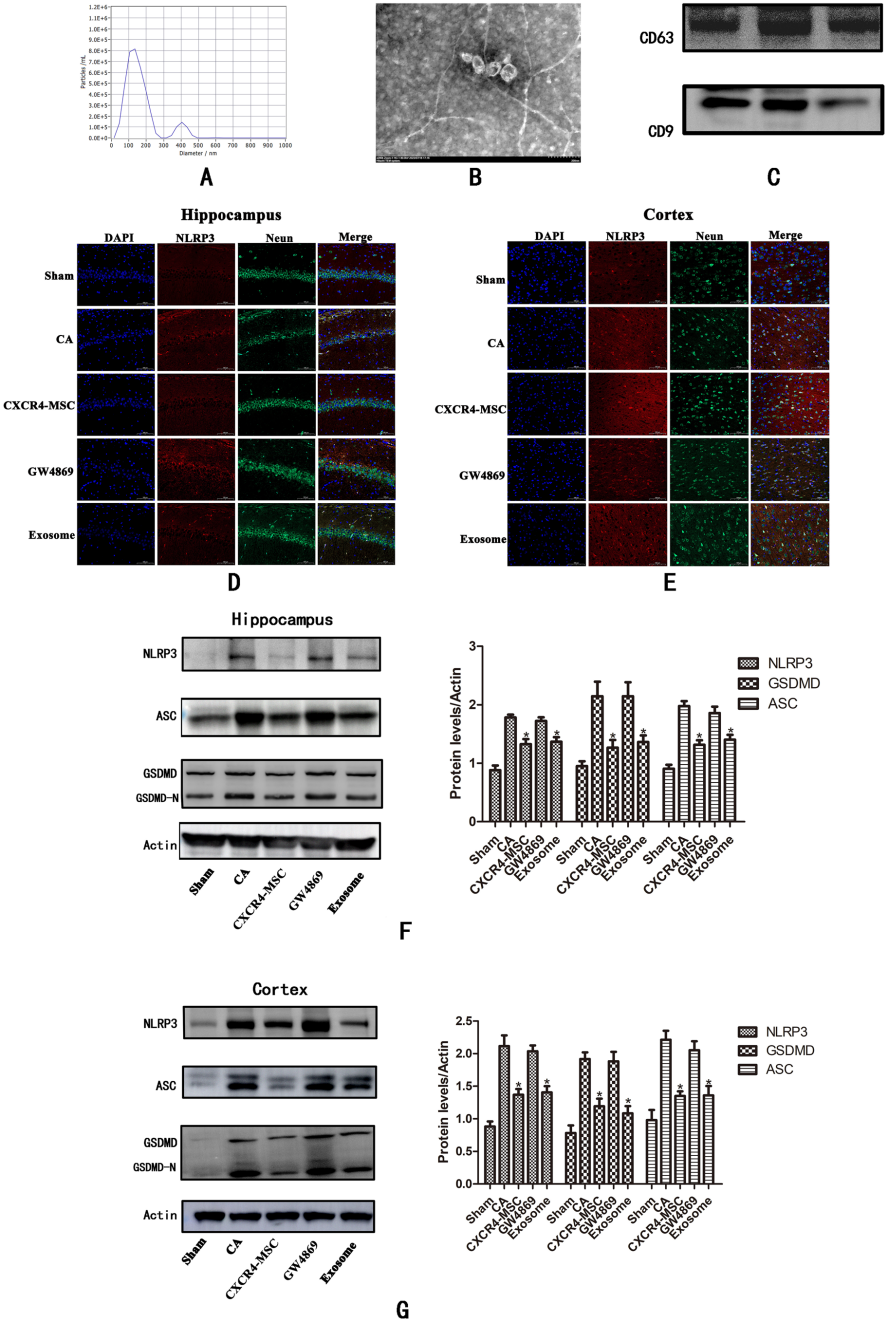
Characterization of CXCR4‐MSC exosomes and expression of pyroptotic protein in each group of rats. (A) The size distribution of exosomes determined by nanoparticle analyzer ranges from 70 nm to 400 nm, with a peak of 110 nm. (B) Electron microscopy showed that the exosomes were round and about 100 nm in diameter. (C) Western blot analysis showed high expression of CD9 and CD63 in exosomes. (D,E) The expression of NLRP3 in hippocampal and cortical neurons was observed by immunofluorescence. (F, G) Expression of pyroptotic protein NLRP3, GSDMD, and ASC in the hippocampus and cortex after resuscitation, *p < 0.05 compared with the CA and GW4869 group.

The extraction of CXCR4‐MSC‐derived exosomes was confirmed through TEM images, which revealed their morphology and an average diameter of approximately 100 nm (Figure 4A). This finding was corroborated by a size distribution curve from a nanoparticle analyzer, indicating a particle size range of 70 to 400 nm, with a peak at 110 nm (Figure 4B). Western blot analysis demonstrated increased expression levels of CD63 and CD9 (Figure 4C), specific markers of exosomes. Subsequently, rats were injected with CXCR4‐MSC‐derived exosomes and their antagonists following CPR. We also specifically labeled neuronal cells (Neun) and pyroptosis‐related protein NLRP3. Observations through immunofluorescence revealed that neurons in the hippocampus and cortex underwent severe pyroptosis after CPR. After the injection of CXCR4‐MSC and the exosomes secreted by CXCR4‐MSC, the expression of pyroptosis‐related protein NLRP3 on neurons was significantly downregulated. The exosome antagonist GW4869 reversed the anti‐pyroptosis effect produced by CXCR4‐MSC Figure 4D,E. However, the GW6789 group of neurons exhibited milder damage, suggesting that in the early stages of recovery, stem cells may improve neuronal injury not only through the secretion of exosomes but also via other possible mechanisms. Western blotting analyses showed that, compared to the CA group, the expression levels of NLRP3 and GSDMD in the nuclear ASC of the CXCR4‐MSC and exosome groups decreased, although the difference between these two groups was not statistically significant. However, after administering exosome antagonists, the levels of pyroptotic protein increased significantly, aligning with those in the CA group Figure 4F,G. These results indicate that CXCR4‐MSC inhibit neuronal pyroptosis in brain tissue following CPR through the secretion of exosomes.

### 2.5 CXCR4‐MSC‐Derived Exosomes Reduce Neuronal Damage and Enhance Behavioral Recovery in Rats Following CPR

**FIGURE 5:**
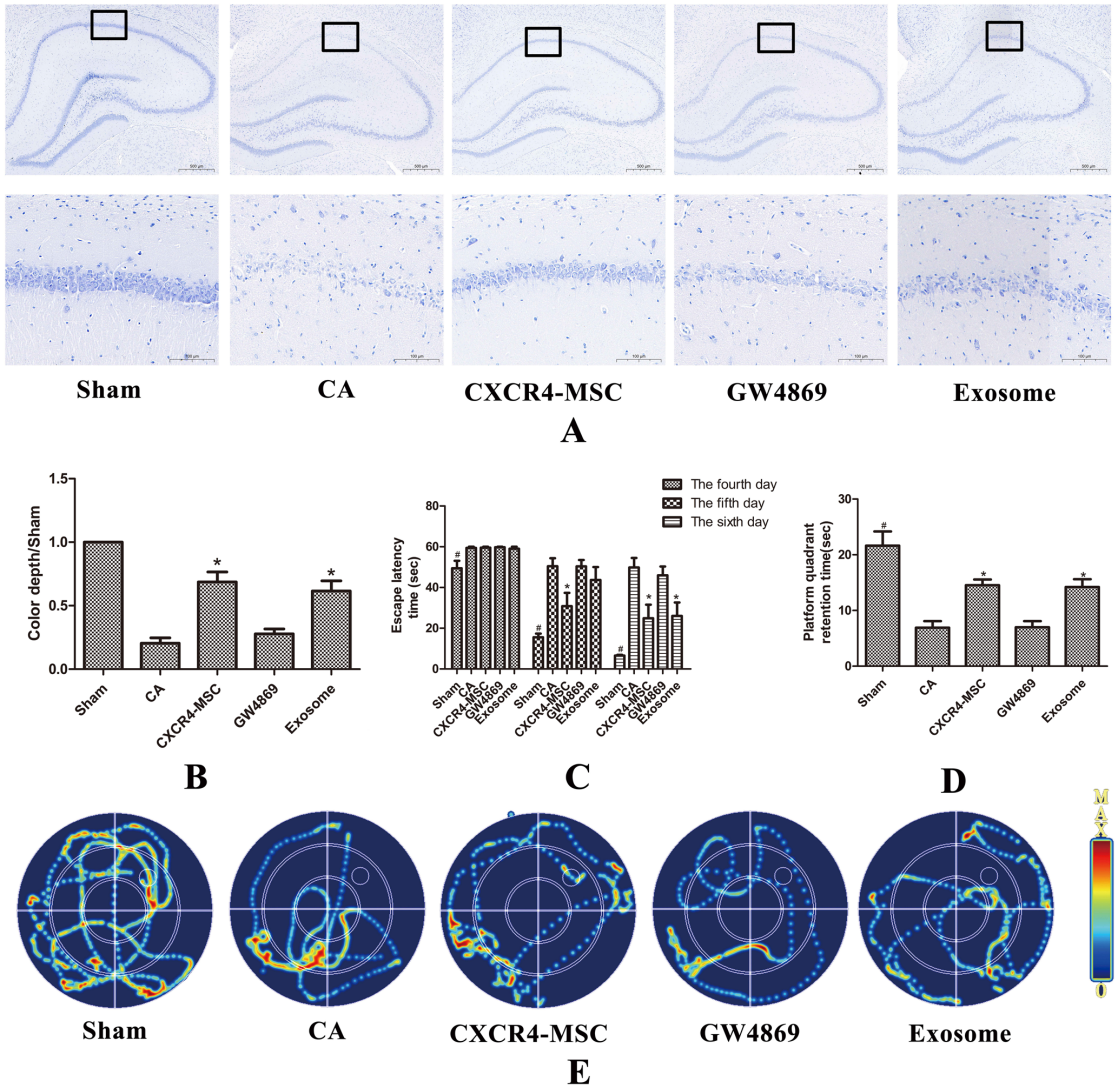
The morphological and behavioral assessment and observation in each group of rats (A) Hippocampus subjected to Nissl staining. (B) Nissl corpuscle ratio in the hippocampus area. *p < 0.05 compared with the CA and GW4869 group. (C) Escape latency time on days 4, 5, and 6 of training after resuscitation. ^#^ *p* < 0.05 compared with other groups, *p < 0.05 compared with the CA group and GW4869 group. (D) Platform quadrant retention time on day 7 of training after resuscitation. ^#^ *p* < 0.05 compared with other groups, *p < 0.05 compared with the CA and GW4869 group. (E) Track map of spatial search on day 7 of training after resuscitation.

The effects of CPR on rat hippocampal neurons were assessed using Nissl staining, revealing neuronal injury. The administration of CXCR4‐MSC‐derived exosomes was found to mitigate neuronal damage; however, the difference between the exosome‐treated group and the CXCR4‐MSC group was not statistically significant (*p* = 0.381). When exosome antagonists were introduced, the therapeutic efficacy of the CXCR4‐MSC group markedly declined (*p* < 0.001). This was evidenced by a significant reduction in the organization of hippocampal cells, a decrease in the number of surviving neurons, and morphological changes in hippocampal neurons that resembled those in the CA group (Figure 5A,B). Furthermore, the Morris water maze experiment demonstrated that, on the 5th and 6th days post‐return of spontaneous circulation (ROSC), the escape latency in the sham operation group was prolonged, and the time spent in the platform quadrant was significantly less than that of the control group. Notably, on the 5th day after resuscitation, the escape latency of the CXCR4‐MSC group was shorter than that of both the CA group (*p* = 0.001) and the exosome antagonist group (*p* = 0.005); there was no statistically significant difference compared with the exosome group (*p* = 0.107). On the 6th day, the exosome group's escape latency was shorter than that of the CA group (*p* < 0.001) and the exosome antagonist group (*p* = 0.001); there was no statistically significant difference compared with the CXCR4‐MSC group (*p* = 0.669) (Figure 5C). Throughout the entire water maze test, there was no statistically significant difference in the platform retention time on the last day between the CXCR4‐MSC group and the exosome group (*p* = 0.167), nor between the exosome antagonist group and the CA group (*p* = 0.942). The target quadrant retention time was longer in both the CXCR4‐MSC group and the exosome group than in the CA group and GW4869 group (Figure 5D,E). This further indicates that in the early stages of recovery, stem cells compensated for the effects of exosome antagonism through other mechanisms. However, as time progressed, exosomes gradually exerted their effects, showing no statistically significant difference compared to stem cells. This suggests that exosomes play an important role in stem cell therapy.

## 3 Method

### 3.1 Isolate, Culture, and Identify Umbilical Cord MSC

The umbilical cord used in this study was obtained from healthy newborns delivered at full term in the Department of Obstetrics and Gynecology at Tangdu Hospital, following the provision of informed consent by the mother and her family. The isolation of primary human umbilical cord MSC was conducted using the tissue block adhesion method. Initially, the umbilical cord was sanitized by immersion in 75% alcohol for 5 min on a sterile operating table to remove any contaminants. Subsequently, the umbilical cord was prepared by eliminating the umbilical artery and vein, and thoroughly cleaning the endothelium of the umbilical vein. The cleaned cord was then cut into 1–2 mm tissue segments, which were carefully placed in a petri dish for cell adhesion. Following cell confluence, typically occurring after 10 to 15 days, cell digestion and subculture procedures were implemented to facilitate cell growth and maintenance. According to the literature, fluorescence quantitative PCR was used to detect the relevant molecules CD44, CD73, CD90, CD105, CD34, and CD45 on the surface of stem cells. Following the fourth passage, MSC showcased their capacity to differentiate into various lineages such as osteoblasts, chondrocytes, or adipocytes. The differentiation experiments were conducted in accordance with the corresponding kits for adipogenic differentiation (UCHX‐D101R, HyCyte, China), chondrogenic differentiation (UBHX‐D203R, HyCyte, China), and an additional kit for adipogenic differentiation (UCHX‐D102R, HyCyte, China) intended for Human Umbilical Cord Marrow Mesenchymal Stem Cells.

### 3.2 Real‐Time Fluorescence Quantification

First, total RNA was extracted from brain tissue using TRIzol reagent (B610409‐0025, Sangon Biotech, Shanghai, China). Subsequently, cDNA was synthesized employing SPARKscript II RT Plus Kit (AG0304, SparkJade, Shandong, China). The real‐time RT‐PCR analysis was conducted using SYBR Mixture (AH0101, SparkJade, Shandong, China) on the LightCycler 480 II instrument (Roche Applied Science), with GAPDH serving as the control for normalization purposes.

### 3.3 Western Blot Analysis

Cells and tissues subjected to the indicated treatments were lysed in buffer (radio‐immunoprecipitation assay buffer (RIPA):PMSF, 100:1) for 30 min, and then centrifuged at 12,000 rpm for 10 min at 4°C. The concentration of proteins derived from the supernatant was determined using the BCA kit (JC‐PD002; Genshare Biological, Beijing, China). Subsequently, 30 μg proteins were separated by sodium dodecyl sulfate‐polyacrylamide gel electrophoresis (SDS‐PAGE) (Genshare Biological JC‐PE001) and transferred to a polyvinylidene fluoride (PVDF) membrane. After the transfer, the membrane was blocked with 5% milk for 0.5 h. Next, the membranes were incubated with primary antibodies β‐actin (D110001; Sangon Biotech, Shanghai, China), CXCR4 (D162693; Sangon Biotech, Shanghai, China), NLRP3 (Cat:381207; zenbio, Chengdu, China), ASC (YT0365; Immunoway, USA), GSDMD (YT7991; Immunoway, USA), CD9 (D364336; Sangon Biotech, Shanghai, China), and CD63 (D264579; Sangon Biotech, Shanghai, China) at 4°C overnight. Following incubation, the membranes were washed and then incubated with horseradish peroxidase conjugated secondary antibodies (AWS0002a; Abiowell, Changsha, China) for 2 h at room temperature. Finally, the membranes were visualized using the ChemiDoc XRS (Bio‐Rad Corporation, USA) chemiluminescence imaging system and quantified using the image acquisition and analysis software Image Lab 3.0.

### 3.4 Transfection

The lentiviral vector expressing the *cxcr4* gene was constructed and purchased from Genko Biotechnology. Following the reagent manufacturer's guidelines, the cells were incubated with the virus overnight. Subsequently, the culture medium was replaced, and purinomycin was employed to screen the stem cells displaying stable expression of the *cxcr4* gene.

### 3.5 CCK‐8 Assay

Cell activity was assessed using the CCK‐8 test following the manufacturer's instructions (CT0001‐A, SparkJade, Shandong, China). To begin, 100 μL cell suspensions (5000 cells/pores) from both MSC and CXCR4‐MSC were aliquoted into 96‐well plates. Every group consisted of 3 holes. The cells were then pre‐incubated in growth medium at 37°C with 5% CO_2_ for 24 h. Subsequently, 10 μL of CCK‐8 solution was added to each well, followed by a 2‐h incubation period in the incubator. Finally, the absorbance change at 450 nm was recorded as an indicator of cell activity.

### 3.6 Migration Assays

Migration assays were conducted using the transwell chamber system equipped with an 8 μm pore filter (PIEP12R48, Millipore). In each upper chamber, serum‐starved MSC and CXCR4‐MSC (2 × 10^5^/well) were seeded, while 500 μL of serum‐free medium, with or without CXCL12 (5 ng/mL, Peprotech), was added to the lower chamber. The experimental groups included the CXCR4‐MSC + CXCL12(+) group, MSC + CXCL12(+) group, CXCR4‐MSC + CXCL12(−) group, and MSC + CXCL12(−) group. Every group had 3 holes. Each experimental condition was replicated three times, and the chambers were incubated at 37°C in a 5% CO_2_ environment for 8 h. Following the incubation period, the cells remaining on the upper surface were carefully removed using cotton swabs. Subsequently, the filter was fixed and stained with 0.1% crystal violet dye. Finally, cells that had migrated to the lower surface were counted under a microscope.

### 3.7 Model of Asphyxial CA

Adult male Sprague–Dawley rats aged 8–10 weeks, provided by the were purchased from HFK Bio‐Technology Co. Ltd. (Beijing, China), were acclimated to the environment for 1 week before the experiment. The experiments were carried out in accordance with the Animal Care and Use Committee of the the Fourth Military Medical University. Every effort was made to limit the number of animals used and minimize their suffering. Following anesthesia with 1% pentobarbital (40–50 mg·kg^−1^), the femoral artery and femoral vein were separated, and a monitoring system was connected to measure blood pressure and pulse (IntelliVue MP50; Philips, Amsterdam, Netherlands). The femoral vein was then reserved for drug administration. Subsequently, the trachea was orally intubated and attached to an animal ventilator (HX‐300S; TECHMAN, Chengdu, China). Mechanical ventilation commenced after blocking the tracheal catheter for 8 min, with ventilation parameters set as follows: tidal volume of 6 mL·kg^−1^, frequency of 80 breaths/min, and a respiratory ratio of 1:1. Adrenalin (0.02 mg·kg^−1^) and sodium bicarbonate (1 mmol·kg^−1^) were injected, while thoracic compression was simultaneously initiated at a frequency of 160 beats/min and an adequate amplitude. Return of spontaneous circulation (ROSC) was considered achieved when Mean Arterial Pressure (MAP) reached 50 mmHg and an independent heartbeat was sustained for at least 10 min. Conversely, if MAP remained below 50 mmHg within the 10‐min timeframe, CPR was deemed unsuccessful.

### 3.8 Anamal Group

To explore the therapeutic effects of CXCR4‐MSC, rats were randomly divided into four groups: The sham group only underwent tracheal intubation and femoral artery–vein catheterization; the CA group was subjected to an asphyxial CA model, and 200 μL of PBS was administered intravenously immediately at ROSC; the CXCR4‐MSC group and the MSC group were given 200 μL of PBS containing 1 × 10^^6^ CXCR4‐MSC and 1 × 10^^6^ MSC, respectively, via intravenous injection at ROSC.

To investigate the role of exosomes secreted by CXCR4‐MSC in therapy, rats were randomly divided into five groups. In addition to the sham group, CA group, and CXCR4‐MSC group, there was also an exosome group and the GW4869 group. The exosome group received an immediate intravenous injection of 200 μL PBS containing 1 × 10^^7^ exosomes at the time of ROSC. The GW4869 group involved adding the exosome antagonist GW4869 (52321ES10, Yeasen Biotechnology, China) at a concentration of 15 μM to CXCR4‐MSC, incubating for 24 h, and then collecting the CXCR4‐MSC. Then, 200 μL of PBS containing 1 × 10^^6^ gw4869‐treated CXCR4‐MSC was intravenously injected into the rat at the time of ROSC.

### 3.9 In Vivo Distribution of Transplanted MSC

24 h before cell transplantation, 5 μL of live cell tracer DiR (S0911D, Meilunbio, Dalian, China) was taken. The working solution was diluted in 5 mL DMEM medium and added to CXCR4‐MSC and MSC, respectively. After 4 h, red fluorescence was observed in the cells under an inverted fluorescence microscope. Subsequently, the prepared dosage was injected into the rat (*n* = 3) model after CPR. Cell migration was then evaluated 24 h later using the imaging system in vivo.

### 3.10 Exosome Extraction and Identification

The culture medium of CXCR4‐MSC, cultured with exosome‐free serum, was processed following the guidelines provided in the exosome extraction and purification kit (UR52121, Umibio Biotechnology, China). To assess the morphology and size of the extracted exosomes, transmission electron microscopy (TEM, H‐7100, Hitachi, Tokyo, Japan) and a nanoparticle tracking analyzer (Videodrop, Myriade, Paris, France) were employed. Subsequently, Western blotting was conducted to verify the presence of specific exosome surface markers, such as CD9 and CD63.

### 3.11 Immunofluorescence

To summarize, the paraffin sections of rats (*n* = 5) from each group underwent dewaxing and rehydration after 24 h. The sections were then incubated in a boiling citric acid buffer for 10 min, followed by natural cooling with 0.1% Triton for another 10 min, and subsequently sealed with 3% bovine serum. Next, the sections were incubated overnight at 4°C with primary antibodies against NLRP3 (Cat: 381207, Zenbio, Chengdu, China) and NeuN (1:100, Cat: 660096, Zenbio, Chengdu, China). After this, the sections were incubated in the dark for 2 h with fluorescently labeled DyLight488 (RS23210, 1:500; Immunoway, USA) and DyLight594 (RS23420, 1:500; Immunoway, USA). Following PBS washing, the sections were sealed with DAPI tablets. For negative controls, the staining procedure omitted incubation with either the primary or secondary antibodies. The stained sections were then observed under a laser confocal microscope (Leica, Heidelberg, Germany), and images were captured and analyzed using NIS‐Elements Viewer 4.20.

### 3.12 Morris Water Maze

In the Morris water maze experiment, the rats (*n* = 10) from each group were evaluated using the TM‐vision behavioral experimental system (Taimeng, TM‐Vision). The pool, which was segmented into four quadrants, housed a colorless transparent platform with a diameter of 10 cm, placed centrally and submerged 2 cm beneath the water's surface. On the fourth day following resuscitation, the rats were initially positioned in a quadrant opposite to the platform, and their escape latencies were then documented. Should any of the rats fail to locate the platform within 60 s, they were assisted to the platform and given a 10‐s stay. Over the course of three consecutive days, the rats underwent three training trials daily. The platform was subsequently withdrawn on the seventh day, with the residence time in the designated target quadrant being recorded within a 60‐s window.

### 3.13 Nissl Staining

Paraffin sections of rat brain tissue after the water maze were dewaxed. Following the preparation, Nissl staining was conducted in accordance with the kit instructions (DK0022–3; Leagene Biotech Beijing, China). Subsequently, five brain sections were extracted from each brain of the five rats in each experimental group for analysis. The sections were then examined under a light microscope and evaluated using Image J software version 1.44 for further analysis.

### 3.14 Elisa

24 h following the experiment, the cerebral cortex and hippocampus of each group of rats (*n* = 5) were anesthetized, and the supernatant was obtained subsequent to grinding and centrifugation. The samples were then diluted as per the instructions provided in the IL‐1β(D731007‐0048, Sangon Biotech, Shanghai, China) Elisa kits. Subsequently, the samples, standard products, enzyme‐labeledIL‐1β enzyme‐labeled IL‐1β were added in sequence and left to incubate at 37°C. Following the incubation period, the plate was washed, and TMB was applied for color development over a 15‐min duration. The reaction was stopped by introducing a termination solution, and the absorbance values were measured at 450 nm utilizing a full‐wavelength microplate reader. Finally, a standard curve was constructed to determine the concentrations of IL‐1β in the sample.

### 3.15 Statistical Analysis

The data were analyzed using a one‐way analysis of variance followed by Student–Newman–Keuls test for pairwise comparisons, and the success rate of rat resuscitation was evaluated using the χ^2^ test. Statistical analyses were conducted using SPSS 17.0 software, and results with *p* < 0.05 were deemed statistically significant. Data were expressed as mean ± standard deviation ($\bar{x}$ ± s).

## 4 Discussion

MSC can be isolated from a variety of tissues and organs. We infected human umbilical cord MSC with CXCR4‐carrying lentivirus, resulting in a significant up‐regulation of *cxcr4* expression at both mRNA and protein levels in human umbilical cord MSC. Interestingly, the infected human umbilical cord MSC did not experience any alterations in cell cycle or proliferation ability. We observed that injection of human umbilical cord mesenchymal stem cells modified by the *cxcr4* gene can increase the number of stem cells in rat brain tissue after CPR. These genetically modified stem cells significantly ameliorated nerve damage, leading to improvements in learning and memory functions in the test subjects. The neuroprotective effects of CXCR4‐MSC are attributed to the exosomes they secrete, which inhibit neuronal pyroptosis.

In a rat model of cerebral ischemia, high‐dose stem cell transplantation significantly enhanced neural function compared with low‐dose transplantation [14], mainly related to stem cell migration to brain tissue [15]. Studies have shown that nano‐iron oxide combined with MSC can increase the amount of brain tissue migration under the action of an external magnetic field, and thus improve the treatment of Alzheimer's disease more effectively [16]. In radiation—or chemically induced oral mucositis mouse studies, MSC that overexpress CXCR2 can enhance their targeting to the site of inflammation, with stronger cell survival, and thus accelerate ulcer healing [13]. The homing efficiency and survival time of stem cells in vivo are the key factors that directly affect their therapeutic effect. However, the unique pathological conditions of the injury site, such as oxidative stress and inflammatory stress, can reduce the self‐renewal ability and survival rate of MSC [17]. Our previous experiments have shown a large number of inflammatory factors infiltrating brain tissue after CPR, which may affect the efficacy of MSC in treating brain injury. In our research, we noted that cxcl12 was highly expressed in brain tissue after CPR. Intravenous injection of CXCR4‐MSC can increase the amount of migration to the rat brain after resuscitation, promote behavioral recovery in rats, and improve the therapeutic effect of brain injury. Similarly, in the myocardial ischemia model, bone marrow MSC with high *cxcr4* expression migrated more to the injured site, thus having more effective therapeutic effects [10]. Other studies have shown that CXCR4 overexpression of mesenchymal stem cells does not improve the homing and therapeutic potential of these cells in acute kidney injury, suggesting that the type and severity of kidney injury may influence the homing of modified stem cells [18].

Pyroptosis is an inflammatory form of programmed cell death that plays a role in the onset and progression of numerous diseases [19, 20, 21]. Bone marrow MSC inhibit NLRP3‐mediated neuroinflammation and pyroptosis, providing neuroprotection [22, 23]. In our experimental model, CXCR4‐MSC was observed to reduce the expression of the pyrogenic protein NLRP3 in brain tissue by secreting exosomes. Some special environments such as inflammation and hypoxia reduce the survival rate of MSC [8], such environments can stimulate the release of exosomes [24], through which its secreted exosomes to improve the prognosis of some neurological diseases such as brain injury, stroke, and epilepsy, Alzheimer's disease [25]. Mesenchymal stem cell‐derived exosomes promote cardiac repair after myocardial infarction by targeting miR‐125a‐5p and macrophage M2 polarization [26]. Bone marrow MSC‐derived exosomes inhibit the N6‐methyladenosine modification level of Drp1 by targeting lncRNA‐ZFAS1 to reduce ischemic stroke [27]. Astrocyte‐derived exosomes reduce ischemic neuroinflammation by regulating NLRP3‐mediated pyroptosis via miR‐378a‐5p [28]. Exosomes mediate neurodegeneration by inducing the pyroptosis of developing hippocampal microglia [29]. We have observed that exosomes can directly inhibit neuronal pyroptosis to promote brain recovery. In our experimental model, CXCR4‐MSC reduces the expression of neuronal upper pyroptotic proteins NLRP3, ASC, and GSDMD through secreted exosomes. Our findings indicate that the injection of exosomes secreted by CXCR4 could promote neural recovery after CPR in rats, and there was no statistically significant difference between the effect produced by the injection and that of CXCR4‐MSC. After the use of exosome antagonists, the therapeutic effect of CXCR4‐MSC was significantly decreased. These results indicate that CXCR4‐MSC secretes exosomes, which are the key substances to improve CA prognosis.

In summary, the study on the CA rat model demonstrated that modified stem cells containing the *cxcr4* gene facilitated neural function recovery by increasing the number of brain injury sites. The primary mechanism identified was the regulation of neuronal pyroptosis through the secretion of exosomes. However, the study was limited in scope; regarding the dosage of stem cell injection, we adopted the dose commonly used in the majority of the literature [30], without conducting dose gradient testing. While the research focus was placed on neuronal injury and we specifically investigated neuronal pyroptosis, we did not investigate whether pyroptosis occurs in other central nervous system cells such as glial cells and endothelial cells. It only observed that exosomes derived from CXCR4‐modified MSC inhibit neuronal pyroptosis. It did not investigate the specific mechanisms by which these exosomes exert their inhibitory effects on neuronal pyroptosis.

Primer sequence (5′‐3′) for RT‐qPCR.

**Table:** 

Primer	Sequence (5′‐3′)
CD105 Forward	GAGGCGGTGGTCAATATC
CD105 Reverse	TCTAACTGGAGCAGGAACT
CD45 Forward	CTACTCCATCTAAGCCAACA
CD45 Reverse	TCCACATTCCACATTCTCAT
CD73 Forward	CCTGTTGGTGATGAAGTTG
CD73 Reverse	GGTGGATTGCCTGTGTAA
CD44 Forward	AGAACGAATCCTGAAGACAT
CD44 Reverse	AGATGTAACCTCCTGAAGTG
CD34 Forward	AAGTGACATCAAGGCAGAA
CD34 Reverse	CCAAGACCAGCAGTAGAC
CD90 Forward	CATCGCTCTCCTGCTAAC
CD90 Reverse	GGTGAACTGCTGGTATTCT
CXCR4 Forward	ATCGTCATCCTGTCCTGTT
CXCR4 Reverse	ACGCTCTCGAACTCACAT
CXCL1 Forward	CACTTCAAGAACATCCAGAG
CXCL1 Reverse	TTGAGTGTGGCTATGACTT
CXCL2Forward	GCTCCTCAATGCTGTACT
CXCL2 Reverse	GAGTGGCTATGACTTCTGT
CXCL3 Forward	TCTGCTGCTTCTGCTGAT
CXCL3 Reverse	ATCCTTGAGAGTGGCTATGA
CXCL9 Forward	ATGAAGTCCGTTGCTCTATT
CXCL9 Reverse	TGTAGTGGAATGTGCCTTG
CXCL10 Forward	TGCTGCTGAGTCTGAGTG
CXCL10 Reverse	CAACATGCGGACAGGATAG
CXCL12 Forward	GCATCAGTGACGGTAAGC
CXCL12 Reverse	CAGTTTGGAGTGTTGAGGAT
β‐Actin Forward	GGTCATCACTATCGGCAAT
β‐Actin Reverse	GTGTTGGCATAGAGGTCTT
GAPDH Forward	TATGACAACAGCCTCAAGAT
GAPDH Reverse	AGTCCTTCCACGATACCA

## Ethics Statement

Animal studies were approved by the Committee on Ethics of Animal Experiments of the Fourth Military Medical University. In accordance with the ARRIVE guidelines and the Guide for the Care and Use of Laboratory Animals recommended by the National Institutes of Health, every effort was made to limit the number of animals used and minimize their suffering.

## Conflicts of Interest

The authors declare no conflicts of interest.

## Supporting information


**Figure S1:** Isolation/characterization of human umbilical cord‐derived MSC. A. Human umbilical cord MSC crawled out of 14d tissue blocks were cultured. B. 3rd generation human umbilical cord MSC. C. The MSC showed CD105, CD90, CD73 and CD44, but almost no expression of CD34, CD45. D, E, F. Representative images showing the trilineage differentiation potential of MSC into adipocytes (oil red O), osteocytes (alizarin red), and chondrocytes (alcian blue).

**Figure S2:** A, B. The migration ability of CXCR4‐MSC and AMD3100 group in vivo was detected in brain of rat after CPR. *p = 0.0171；C, D Hippocampus subjected to Nissl staining between CXCR4‐MSC and AMD3100 group. *p = 0.0392.

## Data Availability

Data sharing not applicable to this article as no datasets were generated or analysed during the current study.
