# Supplementary material for: Genetic Modification of Mesenchymal Stem Cell to Overexpress CXCR4 Enhances Treatment Efficacy for Brain Injury After Cardiopulmonary Resuscitation
Source: CNS Neurosci Ther. 2025 Sep 22;31(9):e70621. doi: 10.1111/cns.70621 (PMC12454672; doi:10.1111/cns.70621)
Supplement: Supplementary file 1 — Figure S1: Isolation/characterization of human umbilical cord‐derived MSC. A. Human umbilical cord MSC crawled out of 14d tissue blocks were cultured. B. 3rd generation human umbilical cord MSC. C. The MSC showed CD105, CD90, CD73 and CD44, but almost no expression of CD34, CD45. D, E, F. Representative images showing the trilineage differentiation potential of MSC into adipocytes (oil red O), osteocytes (alizarin red), and chondrocytes (alcian blue). [file CNS-31-e70621-s002.docx]

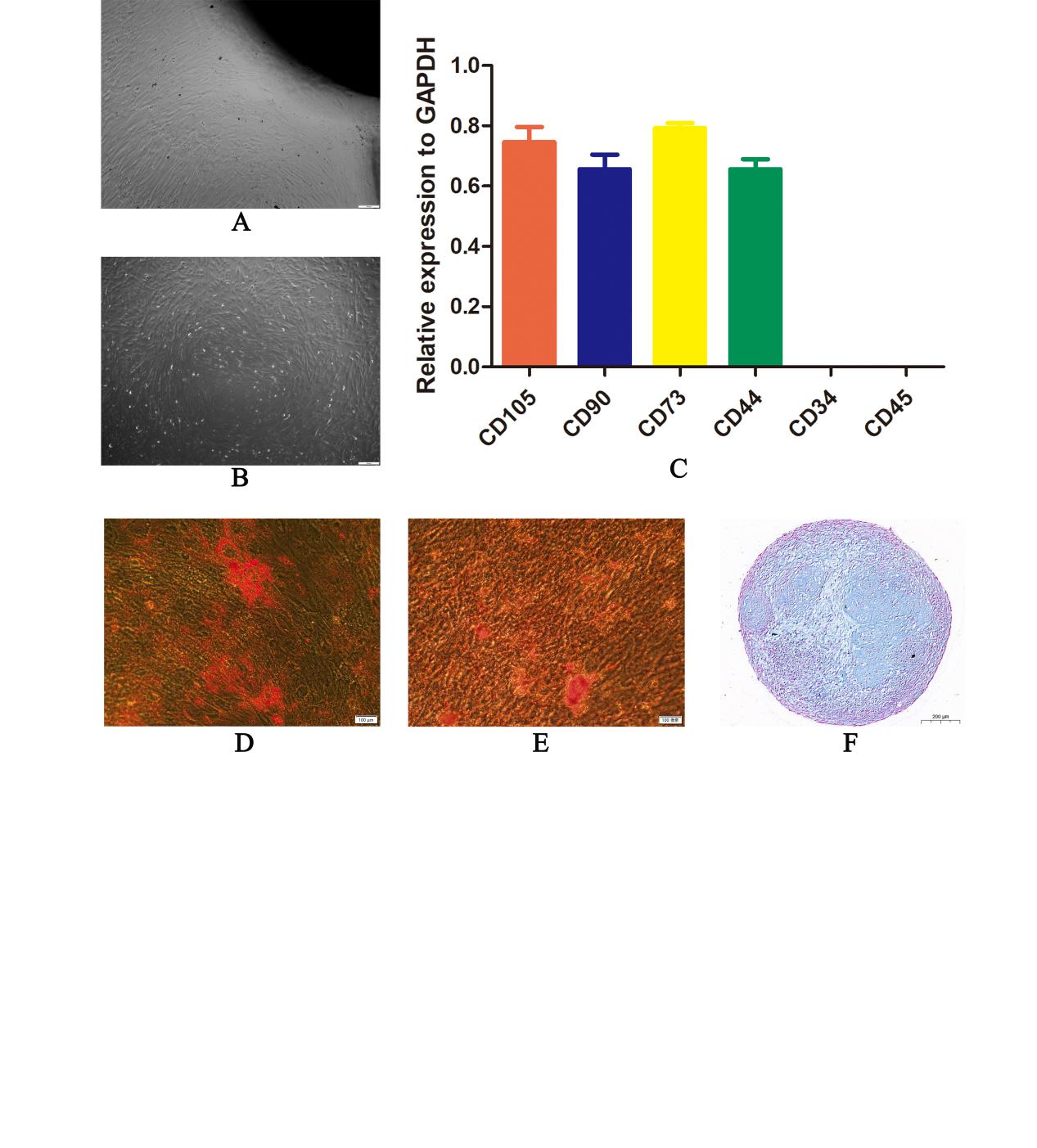


Supplementary Fig.1 Isolation/characterization of human umbilical cord-derived MSC. A. Human umbilical cord MSC crawled out of 14d tissue blocks were cultured. B. 3rd generation human umbilical cord MSC. C. The MSC showed CD105、CD90、CD73 and CD44, but almost no expression of CD34, CD45. D、E、F. Representative images showing the trilineage differentiation potential of MSC into adipocytes (oil red O), osteocytes (alizarin red), and chondrocytes (alcian blue).
