# Supplementary material for: Genetic Modification of Mesenchymal Stem Cell to Overexpress CXCR4 Enhances Treatment Efficacy for Brain Injury After Cardiopulmonary Resuscitation
Source: CNS Neurosci Ther. 2025 Sep 22;31(9):e70621. doi: 10.1111/cns.70621 (PMC12454672; doi:10.1111/cns.70621)
Supplement: Supplementary file 2 — Figure S2: A, B. The migration ability of CXCR4‐MSC and AMD3100 group in vivo was detected in brain of rat after CPR. *p = 0.0171；C, D Hippocampus subjected to Nissl staining between CXCR4‐MSC and AMD3100 group. *p = 0.0392. [file CNS-31-e70621-s001.docx]

。


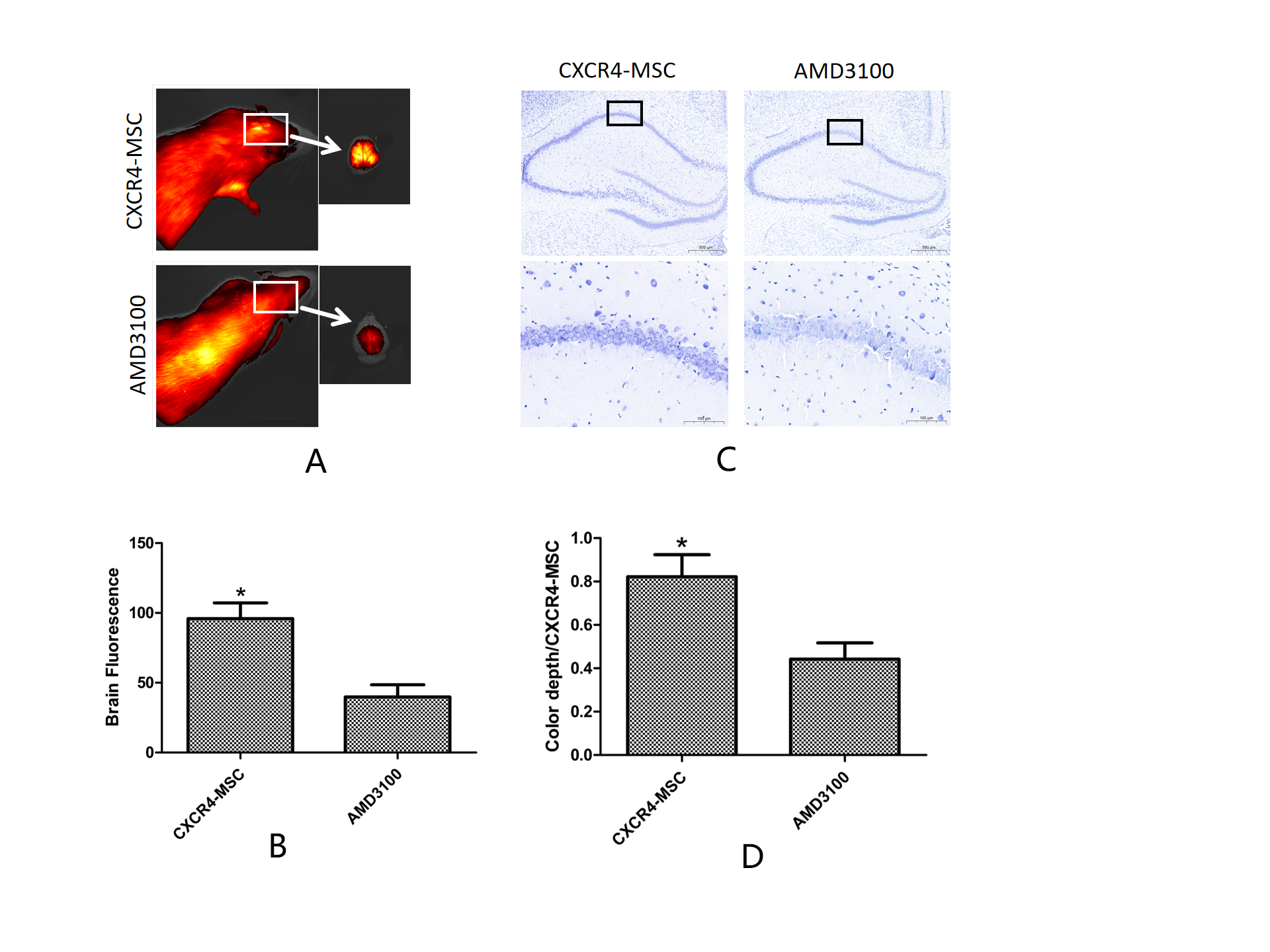


Supplementary Fig 2：A、B. The migration ability of CXCR4-MSC and AMD3100 group in vivo was detected in brain of rat after CPR. **P=* 0.0171；C、D Hippocampus subjected to Nissl staining between CXCR4-MSC and AMD3100 group. **P* = 0.0392
